# Establishment and metabolic analysis of a model microbial community for understanding trophic and electron accepting interactions of subsurface anaerobic environments

**DOI:** 10.1186/1471-2180-10-149

**Published:** 2010-05-24

**Authors:** Lance D Miller, Jennifer J Mosher, Amudhan Venkateswaran, Zamin K Yang, Anthony V Palumbo, Tommy J Phelps, Mircea Podar, Christopher W Schadt, Martin Keller

**Affiliations:** 1 Biosciences and Environmental Sciences Division, Oak Ridge National Laboratory, Oak Ridge, TN 37831, USA; 2 Virtual Institute for Microbial Stress and Survival, LBNL One Cyclotron Road MS 977-152 Berkeley, CA 94720, USA

## Abstract

**Background:**

Communities of microorganisms control the rates of key biogeochemical cycles, and are important for biotechnology, bioremediation, and industrial microbiological processes. For this reason, we constructed a model microbial community comprised of three species dependent on trophic interactions. The three species microbial community was comprised of *Clostridium cellulolyticum*, *Desulfovibrio vulgaris *Hildenborough, and *Geobacter sulfurreducens *and was grown under continuous culture conditions. Cellobiose served as the carbon and energy source for *C. cellulolyticum*, whereas *D. vulgaris *and *G. sulfurreducens *derived carbon and energy from the metabolic products of cellobiose fermentation and were provided with sulfate and fumarate respectively as electron acceptors.

**Results:**

qPCR monitoring of the culture revealed *C. cellulolyticum *to be dominant as expected and confirmed the presence of *D. vulgaris *and *G. sulfurreducens*. Proposed metabolic modeling of carbon and electron flow of the three-species community indicated that the growth of *C. cellulolyticum *and *D. vulgaris *were electron donor limited whereas *G. sulfurreducens *was electron acceptor limited.

**Conclusions:**

The results demonstrate that *C. cellulolyticum*, *D. vulgaris*, and *G. sulfurreducens *can be grown in coculture in a continuous culture system in which *D. vulgaris *and *G. sulfurreducens *are dependent upon the metabolic byproducts of *C. cellulolyticum *for nutrients. This represents a step towards developing a tractable model ecosystem comprised of members representing the functional groups of a trophic network.

## Background

Cultivation of individual microbial species has been at the core of experimental microbiology for more than a century but offers only a glimpse into the collective metabolism, ecology and ecophysiological potential of natural microbial systems. Microbial communities rather than individual species generally control process rates and drive key biogeochemical cycles, including those that determine the transformation of environmental pollutants. While the relatively recent advances in molecular ecology and metagenomic-enabled studies of microbial communities have greatly advanced our understanding of natural and engineered systems, such systems are often not amenable to precise experimental manipulation. Controlled studies of model consortia comprised of multiple species that mediate important biological processes are essential for advancing our understanding of many diverse areas of microbial ecology. Model consortia studies may be especially pertinent to engineered and biotechnology relevant processes including; human and animal environments [1-3], processes relevant to bioremediation and natural attenuation [4-6], bacterially mediated wastewater treatment processes [7,8], and industrial biotechnological applications [9].

In their natural environments, microbial communities are often growth-limited by the availability of carbon and energy [10-12]. For this reason, growth of bacteria in carbon limited continuous-culture systems more closely resembles that in natural ecosystems [13] in contrast to the excess nutrients provided in most microbiological media [13]. Moreover, the steady-state growth condition afforded by continuous-culture systems is more precise and statistically reproducible than the constantly changing physiological states of cells grown under batch culture conditions [13,14]. Therefore these approaches may be favored for model community studies.

Previous studies of mixed cultures in the laboratory focused on understanding the syntrophic growth of sulfate-reducers and methanogens [15,16], competition for nutrients and electron sinks between microorganisms [17-20], and functional community stability [21-23]. However, there is a lack of studies on consortia of microorganisms representing the higher-level trophic interactions based on the archetypical models of the functional groups within a trophic network. For example, an ideal model consortium representing a subsurface anoxic community might comprise a group of microorganisms representing several oxidation-reduction levels. This community would be capable of extracting considerable energy from organic monomers or polymers through various trophic interactions and several terminal electron-acceptor processes, ending with the least thermodynamically favorable process of methanogenesis. In undisturbed and unstimulated groundwater systems the primary carbon sources available may include humic acids and complex mixtures of carbohydrates that derive from the breakdown of vegetation inputs and cell wall constituents, as well as volatile fatty acids derived from the microbial breakdown of such inputs [24,25]. Microbial activity in these systems is thought to be primarily driven by fermenters of complex carbohydrates, with subsequent utilization of fermentation products such as acetate, ethanol and other volatile fatty acids by sulfate reducing bacteria (SRB) and ferric iron reducing bacteria (FRB) that oxidize these products [26-30].

As a first step towards developing a model anaerobic and syntrophic community, we sought to use 3 to 4 model organisms to serve as archetypes for the various functional redox groups. All candidate microorganisms have sequenced genomes http://genome.jgi-psf.org/cloce/cloce.info.html[31,32], tractable genetic systems [33-36], and have been previously studied individually or in co-culture in continuous flow systems [37-42]. *Clostridium cellulolyticum *was chosen as the basal organism due the diverse ability of this organism for the fermentation of complex carbohydrate polymers. As it ferments cellobiose, for example, acetate, lactate, ethanol and hydrogen are produced that can potentially be used by other organisms including SRB and FRB. The secondary stage in the chain of nutrient and electron flow was represented by both *Desulfovibrio vulgaris *and by *Geobacter sulfurreducens*, each of which can utilize the metabolites of *C. cellulolyticum*. In this system, *D. vulgaris *and *G. sulfurreducens *were provided with sulfate and fumarate, respectively, as electron-acceptors in order to avoid electron-acceptor competition as well as the precipitates from using ferric iron as an electron-acceptor for *Geobacter*. Both *Desulfovibrio*-like and *Geobacter*-like organisms also represent organisms commonly responsible for the reduction of Uranium, Chromium and other heavy metals as found in contaminated sites [27-30,43,44]. By constructing this consortia from the *a priori *criteria described above, we were also able to quickly refine minimal medium and cultivation conditions. This strategy also enables the future development and application of analytical methods that take full advantage of genome enabled tools to characterize and track consortia dynamics at the molecular level.

The goals of this study were to; 1) develop a stable microbial consortia in continuous flow systems that could be used for physiological and functional genomic studies in tractable and manipulable experiments, 2) to develop and apply analytical methods for quantifying the community members and monitoring individual as well as community metabolism, and 3) to build a simple metabolic model of the community. Here we present analysis of a stable consortium comprised of *C. cellulolyticum*, *D. vulgaris*, and *G. sulfurreducens *for understanding trophic interactions in anaerobic subsurface environments.

## Results and Discussion

### Tri-culture inoculation and metabolite monitoring reveals limiting nutrients

**Figure 1:**
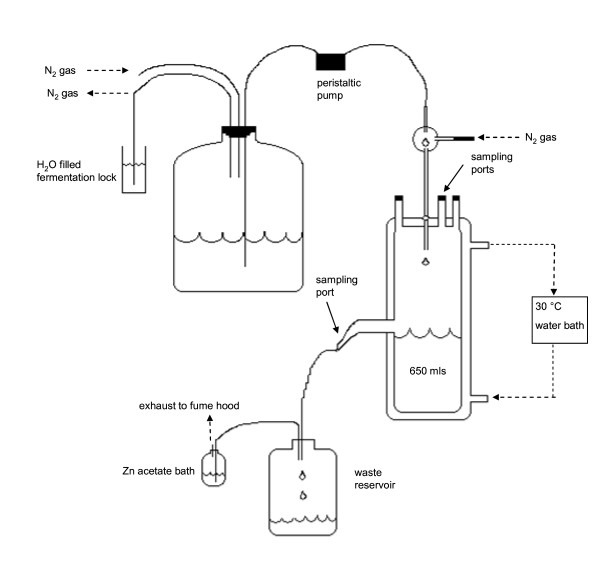
**Chemostat setup**. Schematic diagram illustrating the experimental setup. See text for details.

**Figure 2:**
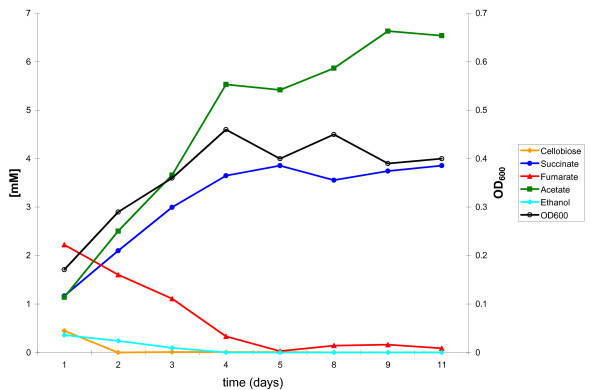
**Metabolic monitoring of the three species community**. HPLC analysis revealed the metabolite flux of the consortia. Cellobiose levels were reduced and acetate levels increased as the optical density, OD_600_, of the culture increased.

Two custom built continuous culture vessels as described in the Materials and Methods section and shown in Figure 1 were each inoculated with 50 ml of a previously grown three species community culture comprised of *C. cellulolyticum*, *D. vulgaris*, and *G. sulfurreducens *with cell numbers and ratios similar to those described here as determined by qPCR that was grown under the same continuous flow conditions. In order to determine the basic metabolic interactions between the three species within this community as it reached steady state, the vessels and the metabolites were monitored. Samples were collected daily from the bioreactor outflow. The OD_600 _of the culture peaked on day 4 at ~0.5 before stabilizing at 0.4 ± 0.03 (Figure 2). The pH remained stable between 7.0 and 7.2 for the course of the experiment without the need for pH control (data not shown). Samples (10 ml) were stored at -20°C for subsequent qPCR analysis, while identical samples (0.5-1 ml) were stored at -20°C for subsequent GC/MS and or HPLC metabolite analysis. The results, shown in Figure 2, were similar to that achieved by a second replicate co-culture grown simultaneously, as well as to six other continuous culture experiments conducted over a 12 month period (data not shown).

In all co-cultures, the 2.2 mM cellobiose decreased to less than 0.5 mM within 2 days and thereafter rarely exceeded 0.1 mM (Figure 2 and Additional File 1). This was different than in preliminary continuous culture experiments where non-steady state "upsets" occurred that were often associated with sporulation of *C. cellulolyticum*. In these cases, the concentration of cellobiose reached up to 2 mM for three or more days until a new steady state approached. Cellobiose fermentation resulted primarily in the production of acetate and CO_2 _at steady state. While quantifiable CO_2 _was within the nitrogen gas flushed across the vessel headspace and exiting the vessel, hydrogen remained below the 0.3 μM detection limit. The concentrations of these compounds stabilized as the culture reached a stable optical density of ~0.4. Ethanol was also occasionally detected at trace amounts.

*D. vulgaris *likely utilized H_2 _and ethanol as the electron donors for sulfate-reduction while acetate likely provided a carbon source. Acetate also provided a carbon and energy source for *G. sulfurreducens *as it used the 5 mM fumarate as an electron-acceptor and produced succinate. The complete removal of fumarate with remaining acetate in solution indicated that the electron-acceptor limited the metabolism and growth of *G. sulfurreducens*. However, in other three species community culture experiments under continuous flow conditions, when > 5 mM fumarate was provided, an "upset" of the steady-state co-culture often resulted that was associated with, and possibly caused by, the accumulation of succinate (data not shown).

In addition to the HPLC analysis, sulfate depletion was measured using a commercially available kit based on the barium chloride assay [45]. These results demonstrated that *D. vulgaris *depleted 6.1 mM sulfate (out of the 8 mM supplied) from the medium by sulfate reduction (Additional File 1). However, sulfate remained in the medium at a concentration of about 2 mM suggesting that *D. vulgaris *was not growth limited by the amount of sulfate available. The abundance of acetate coupled with the availability of sulfate suggests that electron donors were limiting the growth of *D. vulgaris*. Small amounts of hydrogen (< 10 μM) were detected in the culture gas phase as shown in Additional File 1, suggesting its availability for interspecies hydrogen transfer. However, in preliminary experiments using these same reactor conditions, these H_2 _concentrations proved insufficient to support the growth of *Methanococcus maripaludis *over sustained periods at this dilution and gas flushing rate (data not shown). It is possible that a combination of the reactor agitation rate combined with the gas exchange rate decreased the H_2 _partial pressure to a point where the growth of the methanogen was unsustainable.

From the metabolic analysis several conclusions can be drawn about the three species community comprised of C. *cellulolyticum, D. vulgaris*, and *G. sulfurreducens*. Given that cellobiose was virtually exhausted in the culture supernatant, *C. cellulolyticum *was likely growth limited by the availability of cellobiose and not by the dilution rate which was considerably slower than the maximum growth rate observed in monoculture chemostat studies [37,46]. Analysis of the three species community's metabolism coupled with results from a *C. cellulolyticum *single species chemostat fed with a similar medium suggests that *C. cellulolyticum *produced little to no lactate under these conditions (data not shown) in agreement with previous studies [37,46].

#### *Culture composition determined by quantitative PCR*

In order to monitor the cell numbers of the individual species comprising the three species community, a quantitative PCR (qPCR) based method was used to quantify each member of the community over time. Specific primers targeting the 16S small subunit (SSU) rRNA gene for *C. cellulolyticum*, *D. vulgaris*, and *G. sulfurreducens *were designed and are listed in Table 1. The qPCR conditions were optimized as described in the Materials and Methods section.

**Table 1:** Oligonucleotide primers used for qPCR

Primer name	Target Organism	Sequence
DvH-F	*D. vulgaris*	5'-GCGTTAAGCATCCCGCCT-3'
DvH-R	*D. vulgaris*	5'-CATCGAATTAAACCACAT-3'
Geo-F	*G. sulfurreducens*	5'-AGACTTGAGTACGGGAGA-3'
Geo-R	*G. sulfurreducens*	5'-TAGCCGCCTTCGCCACCG-3'
Clos-F	*C. cellulolyticum*	5'-GATGGATACTAGGTGTAG-3'
Clos-R	*C. cellulolyticum*	5'-TTCCTTTGAGTTTCAACC-3'

**Figure 3:**
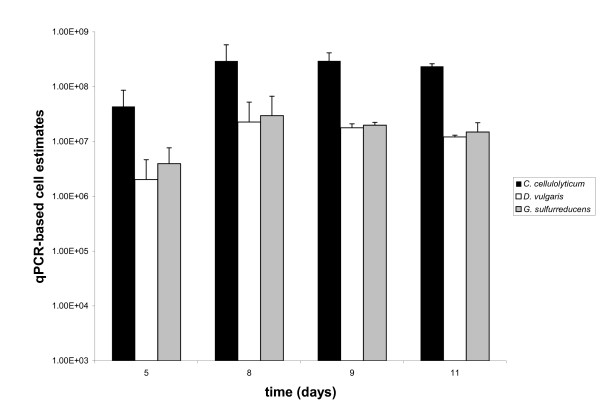
**Cell numbers were quantified using qPCR**. The number of cells of each species present in each of two three species communities was quantified using qPCR. In both communities *C. cellulolyticum *was the dominant member being an order of magnitude greater than *G. sulfurreducens *and *D. vulgaris*.

As expected, the three species community was dominated by *C. cellulolyticum *with *D. vulgaris *and *G. sulfurreducens *present at a level at least an order of magnitude lower (Figure 3). qPCR derived estimates of cell numbers for *C. cellulolyticum *approached approximately 5 × 10^8 ^cells ml^-1 ^(Figure 3 and Table 2), whereas *G. sulfurreducens *and *D. vulgaris *were present in the tri-cultures approximately 10^7 ^cells ml^-1 ^representing roughly an order of magnitude difference. Direct cell counts of these and other tri-cultures as well as the conversion of optical density measurements to cell dry weight were in general agreement that 90% of the cells were *C. cellulolyticum*. qPCR was primarily used to rapidly track the temporal dynamics of the individual species within the cultures on a daily basis, as opposed to being used to provide absolute numbers of each community member.

**Table 2:** Estimated Carbon and e- Recovery of Three Species Community*

	cell counts (× 10^8^)	biomass (mg/L)	C recovered	e^- ^recovered	energy in digestible end products (%)
three species community	5.25	236	93	112	45
*C. cellulolyticum*	*4.6*	*210*	*104*	*120*	*71*
*D. vulgaris*	*0.29*	*13*	*112*	*122*	*7*
*G. sulfurreducens*	*0.36*	*16*	*79*	*83*	*78*

* italicized values are based on the model shown in Figure 5.

### Fluorescent microscopy confirms the presence of each species

**Figure 4:**
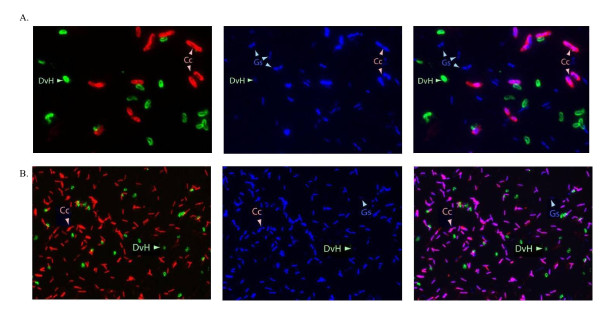
**Fluorescent microscopy confirmed cell ratios**. Fluorescent microscopy using labeled antibodies confirmed the presence of each species in the community. Samples were stained with DAPI and fluorescently labeled antibodies: green for *D. vulgaris *and red for *C. cellulolyticum. G. sulfurreducens *cells were stained blue by DAPI as described in the Materials and Methods section. (A) An artificial mixture of 1:1:1, *C. cellulolyticum*: *D. vulgaris*:*G. sulfurreducens*. Each image was of the same microscopic field. Two separate images taken at different fluorescent wavelengths were merged to form the image on the left showing *C. cellulolyticum *and *D. vulgaris*. The image in the center was taken with DAPI and all cells are visible. The image on the right resulted from merging the fluorescent and DAPI images and reveals the *G. sulfurreducens *cells as stained blue by DAPI. (B) The three species community culture shown in Figure 2 and described in the text was sampled during steady state growth and stained with DAPI and fluorescently labeled antibodies and merged as described above for (A). For (A) and (B) Arrows indicate the same cells of *C. cellulolyticum*, *C.c., D. vulgaris*, DvH, and *G. sulfurreducens, G.s*., imaged under the different conditions.

In order to confirm the presence of all three species in the tri-cultures as well as substantiating the dominance of *C. cellulolyticum*, a fluorescent microscopy based assay that used fluorescent antibodies specific for *C. cellulolyticum *and *D. vulgaris *with DNA specific fluorescent dye 4',6-diamidino-2-phenylindole (DAPI) was employed. Samples of a three species community were collected, fixed with paraformaldehyde, stained with the labeled antibodies and DAPI are shown in Figure 4. Figure 4A shows a similarly stained artificial mixture of cultures of the three individual species combined in an approximate 1:1:1 ratio of cell numbers to demonstrate the sensitivity of the assay to detect cells of each species. *C. cellulolyticum *cells were red, *D. vulgaris *cells were green, and *G. sulfurreducens *cells were blue. The arrows indicate representative cells of each species. Figure 4B shows a sample of the three species community showing the presence of all three species and substantiating the dominance of *C. cellulolyticum *representing nearly 90% of the community with microscopic cell counts averaging 5 × 10^8 ^per ml (Table 2).

### Proposed Carbon and Electron Flow

**Figure 5:**
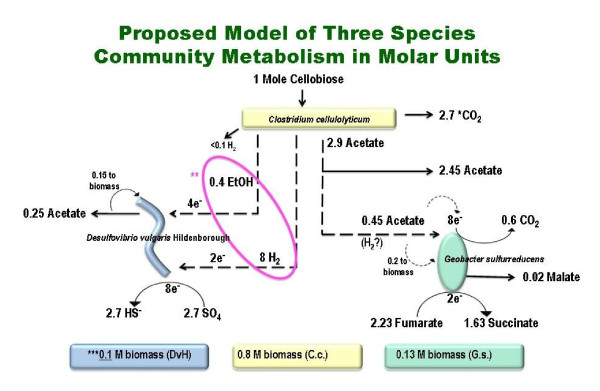
**Proposed model of the metabolic flux and carbon and electron balance of the three member community**. * Values given are in moles. ** Circled electron equivalents could be hydrogen, interspecies electron transfer, or ethanol. See text for details. *** N-moles of biomass determined according to C_4_H_7_O_1.5_N + minerals, 104 gMW (Harris and Adams, 1979). Note: The underlined biomass value (0.1) was used for calculations in Additional File 1.

A model of carbon and electron flow for the three species community was derived from measurements of the three species community steady-state, single culture chemostat experiments, and data from the literature (Figure 5 and Additional File 1 and Table 2). The 640 ml chemostat tri-culture exhibited an OD_600 _of 0.4 with a 236 mg dry weight per liter of biomass. Based on qPCR ratios an approximation was made for each population and used in the model (Table 2 and Figure 5). The overall carbon recovery was estimated at 93% when including cell mass. When modeled for the three populations the values ranged between 79-112%. Similarly, the overall electron recovery was 112% with the individual population models ranging from 83-122%. There was a larger loss of sulfate than readily accounted for causing a modeled electron recovery greater than 120% for *D. vulgaris*, while a loss of carbon in the fumarate-malate-succinate pool resulted in a lower carbon and electron recovery for *G. sulfurreducens*. Because succinate is a readily metabolized end product, 78% of the energy modeled to enter *G. sulfurreducens *was still in some digestible form that could potentially be available for additional microorganisms representing other trophic groups in future experiments. On the other hand, sulfide generation by *D. vulgaris *is of little value for other anaerobic trophic groups. Importantly, 71% of the end products from *C. cellulolyticum *were potentially digestible by other anaerobic trophic groups, and consumption of nearly half of those were evidenced in three-species community described here (Table 2 and Figure 5). The addition of an acetate utilizing methanogen could potentially be envisioned as additional consortia member in this arrangement, however no organisms were readily available that met our other selection criteria (sequenced genome, genetically manipulable, and additionally non-clumping as necessary for efficient chemostat growth).

In the proposed model describing the metabolism of the three species community culture, the culture feed concentration of 2.2 mM cellobiose was completely consumed by the *C. cellulolyticum *with the major end product being 5.93 mM acetate and a similar quantity of CO_2_. A combined 3.3 moles of carbon dioxide was produced by *C. cellulolyticum *and *G. sulfurreducens*, but not by *D. vulgaris *which has an incomplete TCA cycle [32]. Each mole of cellobiose led to 2.7 moles acetate in the supernatant while approximately 0.7 moles of acetate equivalents likely went towards either the electron donating food source of the *Geobacter *or into the biomass of the *Geobacter *and *Desulfovibrio *cells. Hydrogen and ethanol, though generally below detectable limits in tri-culture chemostats, were likely produced by *C. cellulolyticum *and used by *D. vulgaris *to reduce 2.7 moles of sulfate to hydrogen sulfide. The ratio of ethanol and hydrogen available to the sulfate reducer was estimated from the ratio of acetate:ethanol:hydrogen from a pure culture chemostat of *C. cellulolyticum *under the same physical and media conditions (data not shown). However, it was not clear what form of electron equivalents (hydrogen, interspecies electron transfer, or ethanol) was consumed by the sulfate reducer and this could not be distinguished in our measurements so the modeled values are considered preliminary (indicated by the circle in Figure 5). Hydrogen, though abundant in *C. cellulolyticum *pure culture batch experiments, was generally below detectable limits in the three species community, being less than 0.1 mM consumed. *D. vulgaris*, consumed 6.1 mM sulfate (2.7 per mole of cellobiose consumed) leaving behind 2 mM while both hydrogen and ethanol were not detectable suggesting its growth was likely limited by the availability of electron donors. It was possible *D. vulgaris *used fumarate as an electron donor producing succinate and acetate [47] but that was unlikely in the presence of excess sulfate. Fumarate disproportionation would have produced more acetate and succinate and would have resulted in slow growth rates approaching the chemostat dilution rate. Complex interplays of fumarate, malate, succinate, and acetate between the *D. vulgaris *and *G. sulfurreducens *were possible, with the proposed modeled framework (Figure 5) representative of carbon flow and likely electron flow.

*Geobacter sulfurreducens *likely utilized approximately 0.45 moles acetate per mole of cellobiose consumed. Approximately 0.3 moles acetate was modeled as the electron donor producing 0.6 moles CO_2 _with a minor fraction of the acetate incorporated into biomass. While 4.9 mM fumarate was provided to the tri-culture, 2.23 moles of fumarate were transformed per mole of cellobiose consumed. The 2.23 moles of fumarate were reduced to 1.63 moles of succinate with 0.02 moles of malate also detected. Incomplete recovery of the fumarate-malate-succinate couple may be due to some carbon potentially diverted to biomass. *G. sulfurreducens *was electron acceptor limited as verified by its complete removal of fumarate, and being electron acceptor limited likely facilitated electron equivalents being available for sulfate reduction. However, that limitation was forced by an apparent inhibition of the *C. cellulolyticum *whenever succinate approached 10 mM in experiments with elevated fumarate levels (data not shown).

The model of the three species community culture accounts for 236 mg per liter biomass corresponding to 5.25 × 10^8 ^cells per ml. Based upon PCR amplification ratios and cell counts, nearly 80% of the community was comprised of *C. cellulolyticum *with minor contributions by *G. sulfurreducens *and *D. vulgaris *(Figure 5 and Additional File 1). Biomass was ascribed a molecular weight of 104 g/M based on the C_4_H_7_O_1.5_N + minerals formula with the oxidation of said mole requiring 17 electron equivalents of ~ -0.3 mV as described by Harris and Adams 1979 [48]. Accordingly, mass balance determinations accounted for 93% of the carbon and 112% of the electrons available to the tri-culture.

## Conclusions

These results demonstrate that *C. cellulolyticum*, *D. vulgaris*, and *G. sulfurreducens *can be grown in coculture in a continuous culture system in which *D. vulgaris *and *G. sulfurreducens *are dependent upon the metabolic byproducts of *C. cellulolyticum *for nutrients. Moreover, the overall cell densities achieved and maintained under these conditions were appropriate for observing changes in the cell densities resulting from growth or decline from perturbations of nutrients or by stress conditions. Effective methods have been developed to monitor population dynamics and metabolic fluxes of the coculture. This represents a step towards developing a tractable model ecosystem comprised of members representing the functional groups of a trophic network.

Future studies will aim to add additional complexities with the goal of better representing subsurface communities and conditions, as well as responses after perturbing the systems with various stresses (i.e. high salt concentrations, nitrate load, and varying pH conditions) in order to determine how the individual members and the community respond in terms of growth rate and metabolic activity. Efforts are also underway to apply systems biology level approaches, including for example microarrays to determine expression levels of key metabolic genes during the shift to coculture growth as well as during artificially-induced physical and chemical stress.

## Methods

### Strains and Growth Medium

Bacterial strains used in this study were as follows: *Clostridium cellulolyticum *H10 (ATCC 35319), *Desulfovibrio vulgaris *subsp. *vulgaris *Hildenborough NCIMB 8303 [49], and *Geobacter sulfurreducens *[50]. B3M medium as described by Stolyar *et al*. 2007 [15] was modified to support the growth of *C. cellulolyticum *and called B3A. Notably, the buffering agent was changed to 3-(n-morpholino)propanesulfonic acid (MOPS) due to its greater buffering capacity to cope with the fermentation by *C. cellulolyticum *and eliminate the need for continuous pH adjustment of the cultures. B3A medium contained (per liter) 3 g NaCl, 0.5 g MgCl_2_·6H2O, 1 g NH_4_Cl, 0.1 g KCl, 2 g 3-(n-morpholino)propanesulfonic acid (MOPS), and 0.2 mg resazurine added to milli-Q water. The pH was adjusted to 7.2 prior to autoclaving. The following compounds were added from stock solutions after autoclaving to the final concentration shown: 0.2 nM L-alanine, 1 mM CaCl_2_, 2.2 mM cellobiose, 0.2% cysteine, 5 mM fumarate, 5 mM NaHCO_3_, 8 mM Na_2_SO_4, _and 10 mM K_2_HPO_4. _2 ml per liter of a vitamin solution (containing per liter 0.02 g biotin, 0.02 g folic acid, 0.1 g pyridoxine HCl, 0.05 g thiamine HCl, 0.05 g riboflavin, 0.05 g nicotinic acid, 0.05 g calcium pantothenate, 0.05 g p-aminobenzoic acid, 0.01 g vitamin B12, 0.05 g thioctic acid), and 1 ml per liter of a trace minerals solution (containing per liter 0.2 g FeCl_2_·4H_2_O, 0.1 g MnCl_2_·4H_2_O, 0.1 g CoCl_2_·2H_2_O, 0.05 g ZnCl_2_, 0.01 g Na_2_MoO4, 0.005 g H_3_BO_3_, 0.024 g NiCl_2_·6H_2_O, 0.002 g CuCl_2_·2H_2_O, 0.017 g Na_2_SeO_3_·5H_2_O, 0.020 g Na_2_WO_4_·2H_2_O, 1.5 g nitrilotriacetic acid, 0.1 g MgCl_2_·6H_2_O, 1 g CaCl_2_·2H_2_O) was also added after autoclaving.

### Reactor Operation

Two replicate custom built anaerobic glass fermentation vessels (Allen Glass, Boulder, CO) with working volumes of approximately 650 ml were filled with B3A medium (Figure 1). The fermentation vessels were fed medium from the same carboy by individual peristaltic pumps set to deliver media at a flow rate of 0.34 ml min^-1 ^(Figure 1) which was equivalent to a dilution rate of 0.03 h^-1^. The headspace of the 19 L carboy was flushed with N_2 _at ~10 ml min^-1 ^keeping an inert blanket over the medium. Each fermentation vessel was constantly stirred via a magnetic stir bar and anaerobic conditions were maintained by a constant flow of nitrogen gas (49 ml min^-1^) through the medium inlet tube. Sparging the inlet drip-tube proved instrumental in reducing biofilm development in the medium dispensing system and allowed for the prevention of microbial contamination in the sterile medium carboy over four of weeks of operation. The temperature was maintained constant at 30°C ± 2°C by circulating water through the water jackets of each fermentation vessel via a recirculating water bath. Spent culture fluid was allowed to drain out of the vessel overflow vent into a closed collection vessel at the same rate as the replenishing medium thereby maintaining a constant volume. Gas exited the fermentation vessel in the same manner and the collection vessel off gas was passed through an acidified Zn-acetate solution (1% mass to volume) in order to remove hydrogen sulfide before being vented into a chemical fume hood. Gas samples were taken with needles and syringes through ports at the top of the vessels that were sealed with butyl rubber bungs. Liquid samples were taken from the media overflow tubing.

### Genomic DNA Isolation

Total genomic DNA was isolated from the bacterial co-cultures by using the Wizard Genomic DNA purification kit (Promega) according to the manufacturer's protocol with slight modifications. Briefly, 10 ml of co-culture samples were harvested and resuspended in 520 μl of 50 mM EDTA. The cells were further treated with 30 μl of 100 mg/ml lysozyme and incubation at 37°C for 30 minutes followed by addition of 10 μl of 10 mg/ml proteinase K and further incubation at 37°C for 30 minutes. Cell lysis and RNase treatment were performed according to the manufacturer's recommendations. DNA was precipitated with a 0.6 volume of isopropanol, and dissolved in 100 μl TE buffer. The concentration and purity of both DNA and RNA samples were determined by spectrophotometric ratio assay at 260 nm and 280 nm using a Nanodrop spectrophotometer.

### Quantitative Polymerase Chain Reaction (qPCR) Assay

A qPCR assay was employed to monitor the population dynamics of individual bacterial species in the co-culture. Specific primers targeting 16S rRNA genes to track the abundance of individual species in the co-culture via qPCR were designed (Table 1). All assays were performed with the CFX96™ Real Time Detection System (Bio-Rad, Herculus, CA). The fluorescent intensity of SYBR green I, a double-stranded DNA specific dye, was monitored at the end of each extension step, and copy numbers of the target DNAs were estimated by the threshold cycles according to a standard curve. Standard curves were constructed for each organism using their respective genomic DNA and taking into account known genome sizes and copy number. The PCR amplifications were performed in microtiter plates as 30 μl reactions containing the appropriate primers at a final concentration of 0.4 μM, 0.5 μl of the DNA extract, and SYBR green supermix (Bio-Rad, Herculus, CA). Amplification was accomplished by incubating the PCR mixture at 96°C for 15 s, 55°C for 30 s, and 72°C for 30 s for 45 cycles. Melting curve generation followed the amplification, starting at 55°C, with 0.5°C increments at 10 second intervals. For each time point, there were 3 biological replicates and 3 technical replicates in the same plate. qPCR standard curves were constructed using serial dilutions of total genomic DNA for all three bacterial The following equation was used to calculate the number of 16S rRNA gene copy numbers in a known amount of DNA:

where amount is total amount of DNA in ng and length is the total length of DNA in bp. To obtain the 16S rRNA genes copies per ml, the gene copy numbers obtained from the standard curves was multiplied by the total volume of extracted DNA and divided by the volume of sample from which the DNA was extracted and the number of 16S rRNA gene copies for each organism (eight copies for *C. cellulolyticum*, five copies for *D. vulgaris *and two copies for *G. sulfurreducens*).

### Metabolite Analysis

Filtered supernatants were acidified with 200 mM sulfuric acid (giving a final concentration of 5 mM) before injection into a Hitachi Lachrom Elite HPLC system (Hitachi High Technologies, USA). Metabolites were separated on an Aminex HPX-87H column (BioRad Laboratories) under isocratic temperature (40°C) and flow (0.5 ml/min) conditions then passed through a refractive index (RI) detector (Hitachi L-2490). Identification was performed by comparison of retention times with known standards. Quantitation of the metabolites was calculated against linear standard curves. All standards were prepared in uninoculated culture media to account for interference of salts in the RI detector.

Gases were collected from the fermenter vessel headspace via 5 ml syringes and stored at room temperature in 10 ml anaerobic serum bottles from which 5 ml of gas was removed before being analyzed on an Agilent 6850 gas chromatograph (Agilent Technologies, USA) equipped with a thermal conductivity detector (TCD). All gas analytes were separated on an HP-PLOT U column (30m × 0.32 mm × 0.10 um film) (J&W Scientific, Agilent Technologies, USA). Two HP-PLOT U columns were joined together for a total length of 60 m for optimized separation. Samples for carbon dioxide and hydrogen sulfide measurements were injected into a 185°C split-splitless injector with the split ratio set to 3:1 and isocratic oven (70°C) and helium carrier flow (5.1 ml/min). The detector had 10 ml/min helium makeup flow at 185°C, with the detector filament set for positive polarity. Samples to detect hydrogen concentrations were injected into a 185°C split-splitless injector with a split ratio of 3:1 and isocratic oven (180°C) and nitrogen carrier flow (3.5 ml/min). The detector had 10 ml/min nitrogen makeup flow at 185°C with the detector filament at negative polarity. Peak identifications were performed by comparison with known standards. Quantification of each compound was calculated against individual linear standard curves. Henry's Law was used to calculate the solubility of the gases in the media. For carbon dioxide, a modified Henry's Law calculation accounting for the chemical reactivity of the gas was used to determine the amount of gas in solution [51].

Sulfate concentrations were measured using the Sulfaver 4 kit according to Hach Company's instructions. Aqueous hydrogen sulfide was determined by a colorimetric method developed by Pachmayr and described by Brock *et al*. 1971 [52].

### Fluorescence Microscopy and Direct Cell Counts

Cells were fixed in 4% paraformaldehyde for 20 min at room temperature and washed 3 times in phosphate buffered saline (PBS; 137 mM NaCl, 10 mM phosphate, 2.7 mM KCl [pH 7.4]) and resuspended in PBS. The fixed cells (2 to 5 × 10^6 ^cells) were collected on a 0.2-μm black polycarbonate filter (Millipore, Isopore GTPB 02500), and the cells on the filter were transferred to 0.1% gelatin coated slides which contained 5 microliters of water by applying a vacuum for 5 minutes to transfer the cells to the slides [53]. The cells were incubated with fluorophore conjugated polyclonal antibodies FITC for *D. vulgaris *and Rhodamine for *C. cellulolyticum *for 30 min at room temperature, washed with PBS three times, and subsequently were stained with DAPI (4',6'-diamidino-2-phenylindole) 3 μM for 15 minutes. *SlowFade*^® ^Gold from Invitrogen was applied to the slides and the slides were mounted on a Zeiss AX10 microscope. Images were taken by a black and white AxioCam MRm digital camera (Carl Zeiss, Inc.) and then colorized to the appropriate color and merged using photo editing software. Microscopic direct counts of cells were performed using a Petroff Hausser Counting Chamber using a Zeiss Axioskop 2 plus microscope.

### Carbon and Electron Balance and Metabolic Modeling

The metabolic model of the three species community including the carbon and electron balance was designed based on the replicate fermenter steady-state and single culture chemostats and was complemented by batch culture experiments and data from the literature.

For a 640 ml culture with an OD_600 _of 0.4, the biomass was 236 mg dw/L based on a cell dry weight biomass of 590 mg dw/L for a *C. cellulolyticum *culture with an OD_600 _of 1.0 and 1.3 × 10^9 ^cells/ml. The 236 mg per liter biomass corresponded to 5.25 × 10^8 ^cells per ml. Fractions of the specific populations were based upon PCR amplification ratios and cell counts. Biomass was ascribed a molecular weight of 104 g/M based on the C_4_H_7_O_1.5_N + minerals formula with the oxidation of said mole requiring 17 electron equivalents of ~ -0.37 mV as described by Harris and Adams 1979 [47].

Carbon and electron balances in Tables 2 and 1 were based on the model (Figure 5) and analytics, accomplished by comparing carbon inputs with products. The electron balance was based on electron equivalents of inputs compared to electron equivalents of products, including biomass as described above. The fraction of energy available in digestible end products was based on the number of electron equivalents and their energies of all substrates as compared to the energy of the electron equivalents in readily digestible end products such as acetate, succinate, ethanol or hydrogen but excluding biomass or sulfide.

## Authors' contributions

LM performed the continuous culture experiments, analyzed the data, and drafted the manuscript. JM performed the metabolic analysis. AV performed the quantitative PCR analysis. ZY performed the fluorescent antibody experiments. AP, TP, MP, CS, and MK conceived of the study, and participated in its design and coordination. All authors read and approved the final manuscript.

## Supplementary Material

Additional file 1 **Carbon Flow Table**. A table showing the measured and modeled carbon flow of the three species community and populations. Click here for file
