# Supplementary material for: Establishment and metabolic analysis of a model microbial community for understanding trophic and electron accepting interactions of subsurface anaerobic environments
Source: BMC Microbiol. 2010 May 24;10:149. doi: 10.1186/1471-2180-10-149 (PMC2906461; doi:10.1186/1471-2180-10-149)
Supplement: Additional file 1 — Carbon Flow Table. A table showing the measured and modeled carbon flow of the three species community and populations. [file 1471-2180-10-149-S1.DOC]

**Additional File 1 - Measured and Modeled Carbon Flow of the Three Species Community and Populations**

Input electron donor End products Electron acceptors

cellobiose acetate ethanol H_2_ CO_2_ succinate fumarate malate SO_4_

depleted depleted

three species 2.2 5.93 0.01 <0.1 7.26 3.6 0.49 0.04 6.1

community

*C. cellulolyticum 2.2 5.8 0.88 17.6 5.94 - - - -*

*D. vulgaris - 0.55 - - - - - - 6.1*

*G. sulfurreducens - - - - 1.32 3.6 0.49 0.04 -*

* italicized values are based on the model shown in Figure 5.
